# Supplementary material for: Emergent Role of IFITM1/3 towards Splicing Factor (SRSF1) and Antigen-Presenting Molecule (HLA-B) in Cervical Cancer
Source: Biomolecules. 2022 Aug 8;12(8):1090. doi: 10.3390/biom12081090 (PMC9405601; doi:10.3390/biom12081090)
Supplement: Supplementary file 1 [file biomolecules-12-01090-s001.zip › biomolecules-1822071-supplementary.pdf]

Supplementary Information

Maria Gómez-Herranz [1,2,*], Jakub Faktor [2,3], Marcos Yébenes Mayordomo [1,2], Magdalena Pilch [1,2], Marta Nekulova [3], Lenka Hernychova [3], Kathryn L. Ball [1], Borivoj Vojtesek [3], Ted R. Hupp [1,2,3] and Sachin Kote [2,*]

[1] Institute of Genetics and Cancer, University of Edinburgh, Edinburgh EH4 2XU, UK
[2] International Centre for Cancer Vaccine Science, University of Gdańsk, 80-822 Gdańsk, Poland
[3] Masaryk Memorial Cancer Institute, Research Centre for Applied Molecular Oncology, 65653 Brno, Czech
* Correspondence: maria.gomez@ug.edu.pl (M.G.-H.); sachin.kote@ug.edu.pl (S.K.)

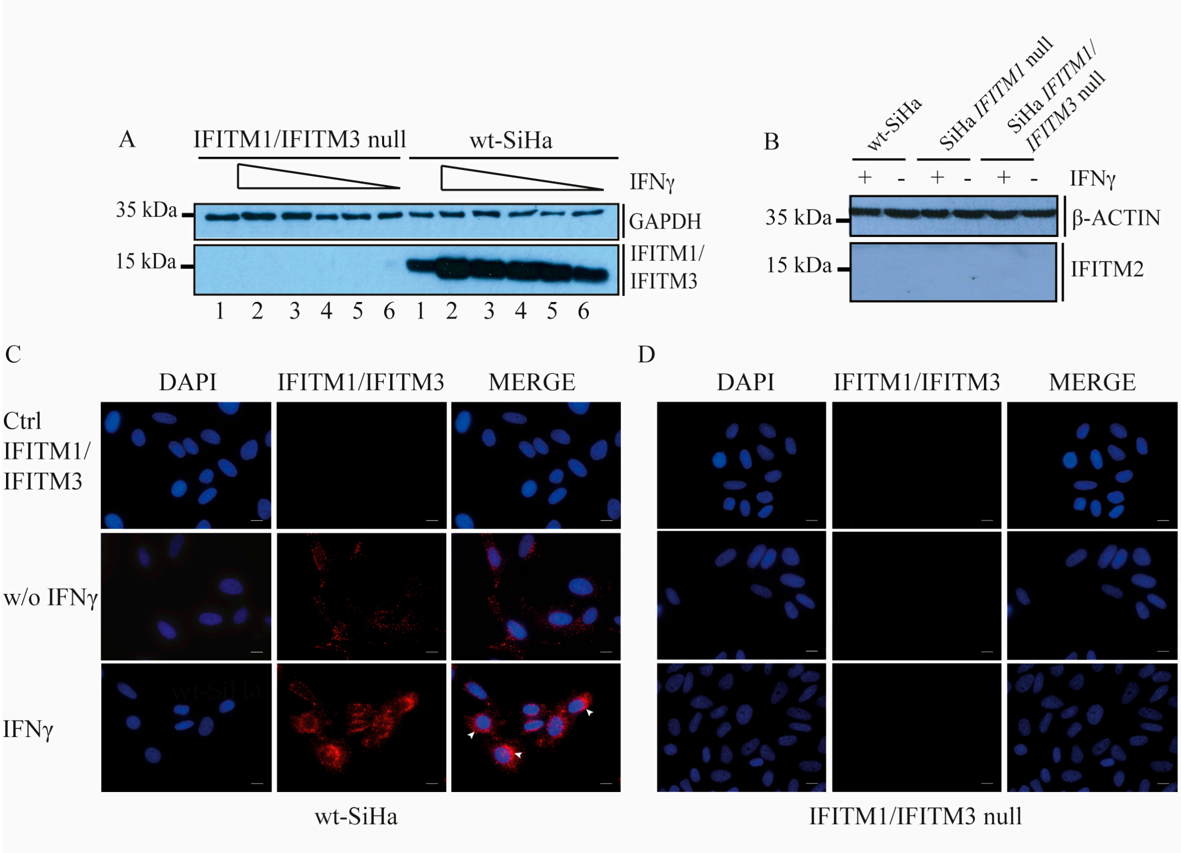

Figure S1. Defining IFITM1/2/3 expression in IFITM1/3-knockout SiHa cells. (A) Dose dependent titration in wt-SiHa and IFITM1/IFITM3 null cells with IFNγ-stimulation for 24 h: nontreated (lane 1), 2000 (lane 2), 1000 (lane 3), 100 (lane 4), 50 (lane 5), and 10 (lane 6) ng/ml. Cells were tested by Western blotting using the IFITM1/IFITM3 antibody for detection (approximately 15 kDa). GAPDH (approximately 35 kDa) was used as a loading control. (B) IFITM2 induction was tested by comparing endogenous protein expression to cells stimulated with 100 ng/ml IFNγ for 24 h. IFITM2 protein expression was not detected despite IFNγ-stimulation. Cells were tested by Western blotting using the IFITM2 antibody for detection (approximately 15 kDa). β-ACTIN (approximately 35 kDa) was used as a loading control. Wt-SiHa cells (C) and IFITM1/IFITM3 null (D) were grown to 80% confluency and fixed with 4% (w/v) paraformaldehyde, permeabilized using 0.25% Triton X-100 and blocked with 3% (w/v) BSA. Immunofluorescence was performed detecting IFITM1/IFITM3 protein in nonstimulated cells (w/o IFNγ) or cells stimulated with 100 ng/ml IFNγ (IFNγ) for 24 h. The negative control (Ctrl IFITM1/3) was performed by solely staining with anti-mouse Alexa Fluor 594 secondary

antibody. Arrowhead colored in white (C; merged column, IFNγ row) indicate the perinuclear distribution of IFITM1/IFITM3 protein in wt-SiHa upon IFNγ stimulation. Scale bar: 10 µm.

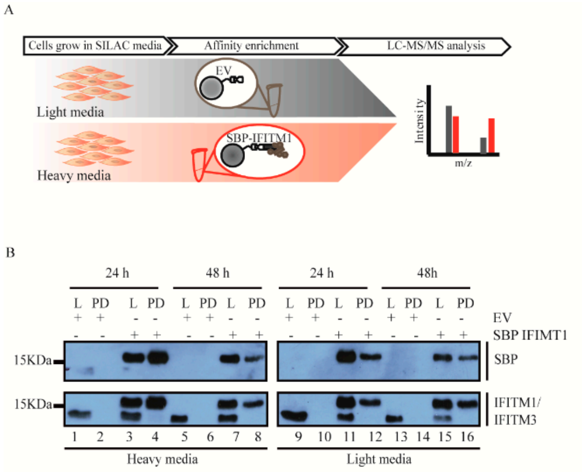

C

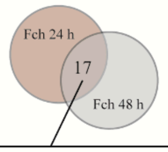

Accession number	Protein name	Fch 24 h	Fch 48 h
Q01130	Serine/arginine-rich splicing factor 2	12.056	0.428
P13164	Interferon-induced transmembrane protein	10.586	3.970
P53999	Activated RNA polymerase II transcriptional coactivator p15	8.211	0.294
P19338	Nucleolin	5.599	0.370
Q01105	Protein SET	4.228	0.170
Q13247	Serine/arginine-rich splicing factor 6	3.752	0.435
Q96EP5	DAZ-associated protein 1	3.466	0.278
Q9P035	Very-long-chain (3R)-3-hydroxyacyl-[acyl-carrier protein] dehydratase	2.789	1.206
Q92945	Far upstream element-binding protein 2	2.710	0.183
P06748	Nucleophosmin	2.127	0.222
Q9BVC6	Transmembrane protein 109	2.022	0.112
Q07955	Serine/arginine-rich splicing factor 1	1.977	0.568
Q9Y2W1	Thyroid hormone receptor-associated protein 3	1.950	0.452
P84103	Serine/arginine-rich splicing factor 3	1.860	0.380
Q9BUJ2	Heterogeneous nuclear ribonucleoprotein U-like protein 1	1.500	0.193
Q01081	Splicing factor U2AF 35 kDa subunit	1.024	0.172

D

SRSF1
Isoform ASF-1 (accession number: Q07955) 24 KDa

```
        10         20         30         40         50
MSGGGVIRGP AGNNDCRIYV GNLPPDIRTK DIEDVFYKYG AIRDIDLKNR
        60         70         80         90        100
RGGPPFAFVE FEDPRDAEDA VYGRDGYDYD GYRLRVEFPR SGRGTGRGGG
       110        120        130        140        150
GGGGGGAPRG RYGPPSRRSE NRVVVSGLPP SGSWQDLKDH MREAGDVCYA
       160        170        180        190        200
DVYRDGTGVV EFVRKEDMTY AVRKLDNTKF RSHEGETAYI RVKVDGPRSP
       210        220        230        240
SYGRSRSRSR SRSRSRSRSN SRSRSYSPRR SRGSPRYSPR HSRSRSRT
```

SRSF2
Isoform 1 (accession number: Q01130) 25.4 KDa

```
        10         20         30         40         50
MSYGRPPPDV EGMTSLKVDN LTYRTSPDTL RRVFEKYGRV GDVYIPRDRY
        60         70         80         90        100
TKESRGFAFV RFHDKRDAED AMDAMDGAVL DGRELRVQMA RYGRPPDSHH
       110        120        130        140        150
SRRGPPPRRY GGGGYGRRSR SPRRRRRSRS RSRSRSRSRS RSRYSRSKSR
       160        170        180        190        200
SRTRSRSRST SKSRSARRSK SKSSSVSRSR SRSRSRSRSR SPPPVSKRES
       210        220
KSRSRSKSPP KSPEEEGAVS S
```

SRSF6
Isoform SRP55 (accession number: Q13247) 39.5 KDa

```
        10         20         30         40         50
MPRVYIGRLS YNVREKDIQR FFSGYGRLLE VDLKNGYGFV EFEDSRDADD
        60         70         80         90        100
AVYELNGKEL CGERVIVEHA RGPRRDRDGY SYGSRSGGGG YSSRRTSGRD
       110        120        130        140        150
KYGPPVRTEY RLIVENLSSR CSWQDLKDFM RQAGEVTYAD AHKERTNEGV
       160        170        180        190        200
IEFRSYSDMK RALDKLDGTE INGRNIRLIE DKPRTSHRRS YSGSRSRSRS
       210        220        230        240        250
RRRSRSRSRR SSRSRSRSIS KSRSRSRSRS KGRSRSRSKG RKSRSKSKSK
       260        270        280        290        300
PKSDRGSHSH SRSRSKDEYE KSRSRSRSRS PKENGKGDIK SKSRSRSQSR
       310        320        330        340
SNSPLPVPPS KARSVSPPPK RATSRSRSRS RSKSRSRSRS SSRD
```

SRSF3
Isoform 1 (accession number: P84103) 19.3 KDa

```
        10         20         30         40         50
MHRDSCPLDC KVYVGNLGNN GNKTELERAF GYYGPLRSVW VARNPPGFAF
        60         70         80         90        100
VEFEDPRDAA DAVRELDGRT LCGCRVRVEL SNGEKRSRNR GPPPSWGRRP
       110        120        130        140        150
RDDYRRRSPP PRRRSPRRRS FSRSRSRSLS RDRRRERSLS RERNHKPSRS
       160
FSRSRSRSRS NERK
```

U2AF1
Isoform 1 (accession number: Q01081) 27.8 KDa

```
        10         20         30         40         50
MAEYLASIFG TEKDKVNCSF YFKIGACRHG DRCSRLHNKP TFSQTIALLN
        60         70         80         90        100
IYRNPQNSSQ SADGLRCAVS DVEMQEHYDE FFEEVFTEME EKYGEVEEMN
       110        120        130        140        150
VCDNLGDHLV GNVYVKFRRE EDAEKAVIDL NNRWFNGQPI HAELSPVTDF
       160        170        180        190        200
REACCRQYEM GECTRGGFCN FMHLKPISRE LRRELYGRRR KKHRSRSRSR
       210        220        230        240
ERRSRSRDRG RGGGGGGGGG GGGRERDRRR SRDRERSGRF
```

Figure S2. Identification of IFITM1 interacting proteins. (A) SILAC workflow describing the stages followed for identifying the IFITM1 binding proteins using SBP-tagged IFITM1 and SBP empty-vector control; stage include cell labelling, transfection, affinity purification using streptavidin beads, elution of the IFITM1 complexes, FASP and LC-MS/MS analysis. (B) An immunoblot of lysates from cells transfected with SBP-tagged IFITM1 or SBP empty vector. Cells were harvested after 24 and 48 h following SBP-IFITM1 or SBP-vector transfection. Transfections were performed in cells grown with heavy and light SILAC media. Lanes were loaded with Lysate [L] and Pull-down [PD]. SBP-tagged IFITM1 was detected in lysates of transfected cells grown in heavy (R6K6) media (lanes 1-8) or light (R0K0) media (lanes 9-16). The transfected SBP-IFITM1 protein was detected in the lysate using either anti-SBP antibody (top panel) or IFITM1/IFITM3-specific monoclonal antibody (lower panel; lanes 3, 7, 11 and 15). SBP-IFITM1 protein was detected by a migration at 19 kDa and the endogenous IFITM1/IFITM3 proteins migrated at a position of 13.9 kDa and 14.6 kDa, respectively. The transfected SBP-IFITM1 protein was detected in the lysate at either 24 or 48 h post-transfection into cells with heavy media (lanes 3 and 7) or light media (lanes 11 and 15). (C) Overlapping of proteins detected by MS from 24 or 48 h post transfection and affinity purification of SBP-IFITM1 including the top proteins enriched in the IFITM1 enrichment (from SI Appendix, Table S1). (D) Identified SRSF peptides in SBP-IFITM1 protein enrichment after affinity purification in isotopically labelled wt-SiHa cells. Heavy isotopically labelled tryptic peptides identified from the SBP-IFITM1 affinity enrichment are highlighted in red for the SRSF family of proteins (SRSF1, SRSF2, SRSF3, SRSF6 and U2AF1). Underlined unique peptides confirm that these proteins are SRSF isoforms reported to shuttle between the cytoplasm and nucleus.

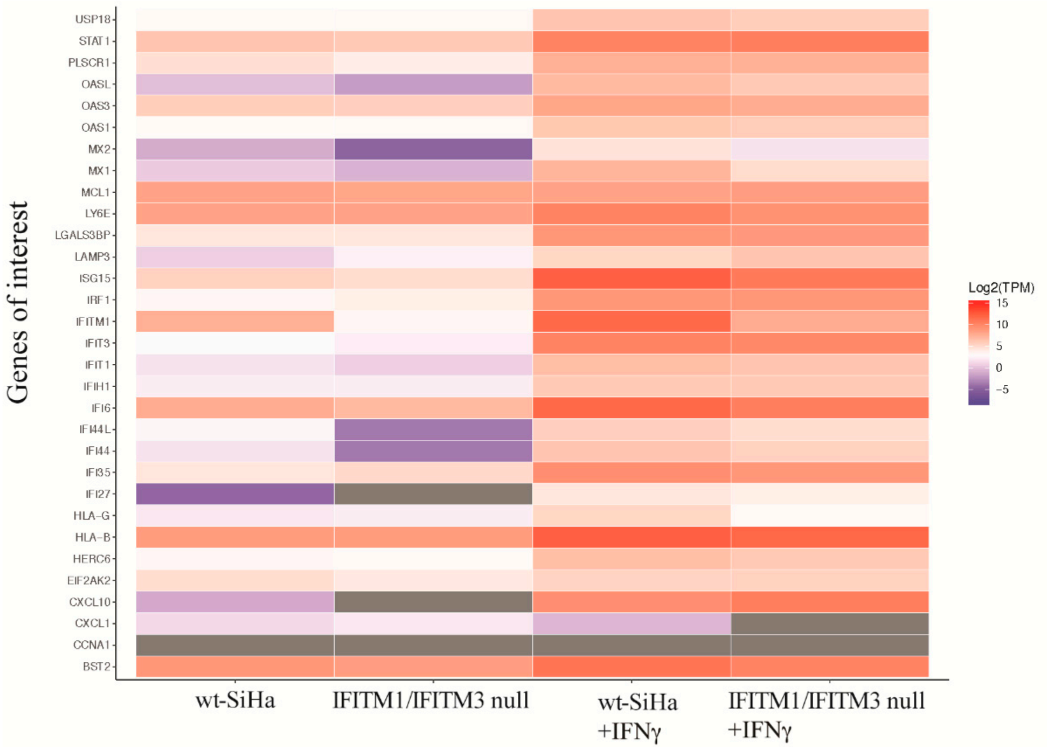

Figure S3. Heat map representation of the mRNA induction of IRDS genes. The x-axis contains the wt-SiHa and IFITM1/IFITM3 null cells nonstimulated or IFNγ-stimulated with 100 ng/ml for 24 h. The y-axis contains transcript expression corresponding to the 31 IRDS genes. Color scale units are log2 (TMP), becoming red when it is highly expressed and purple to blue for nonexpressed and under-expressed values. Values in grey color correspond to genes which TPM was equal to 0. TPM=transcript per million. The heatmap was developed by taking the log2 (TPM) count of the 31 IRDS genes; representation of the genes of interest (IRDS) is extracted from SI Appendix, Table S2. R version 3.5.3 and ggplot2 3.2.1 was to create the heatmaps.

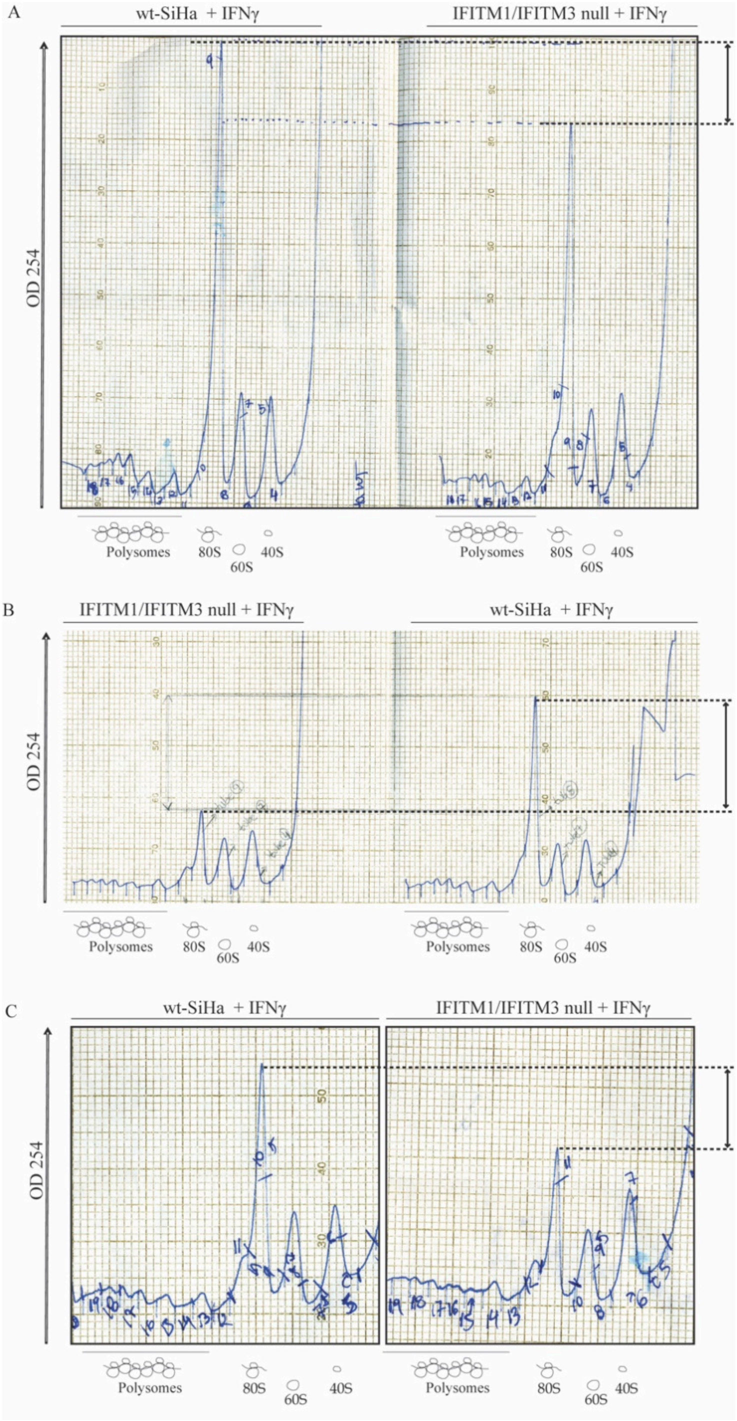
A
wt-SiHa + IFNγ
IFITM1/IFITM3 null + IFNγ
OD 254
Polysomes
80S
60S
40S
B
IFITM1/IFITM3 null + IFNγ
wt-SiHa + IFNγ
OD 254
Polysomes
80S
60S
40S
C
wt-SiHa + IFNγ
IFITM1/IFITM3 null + IFNγ
OD 254
Polysomes
80S
60S
40S

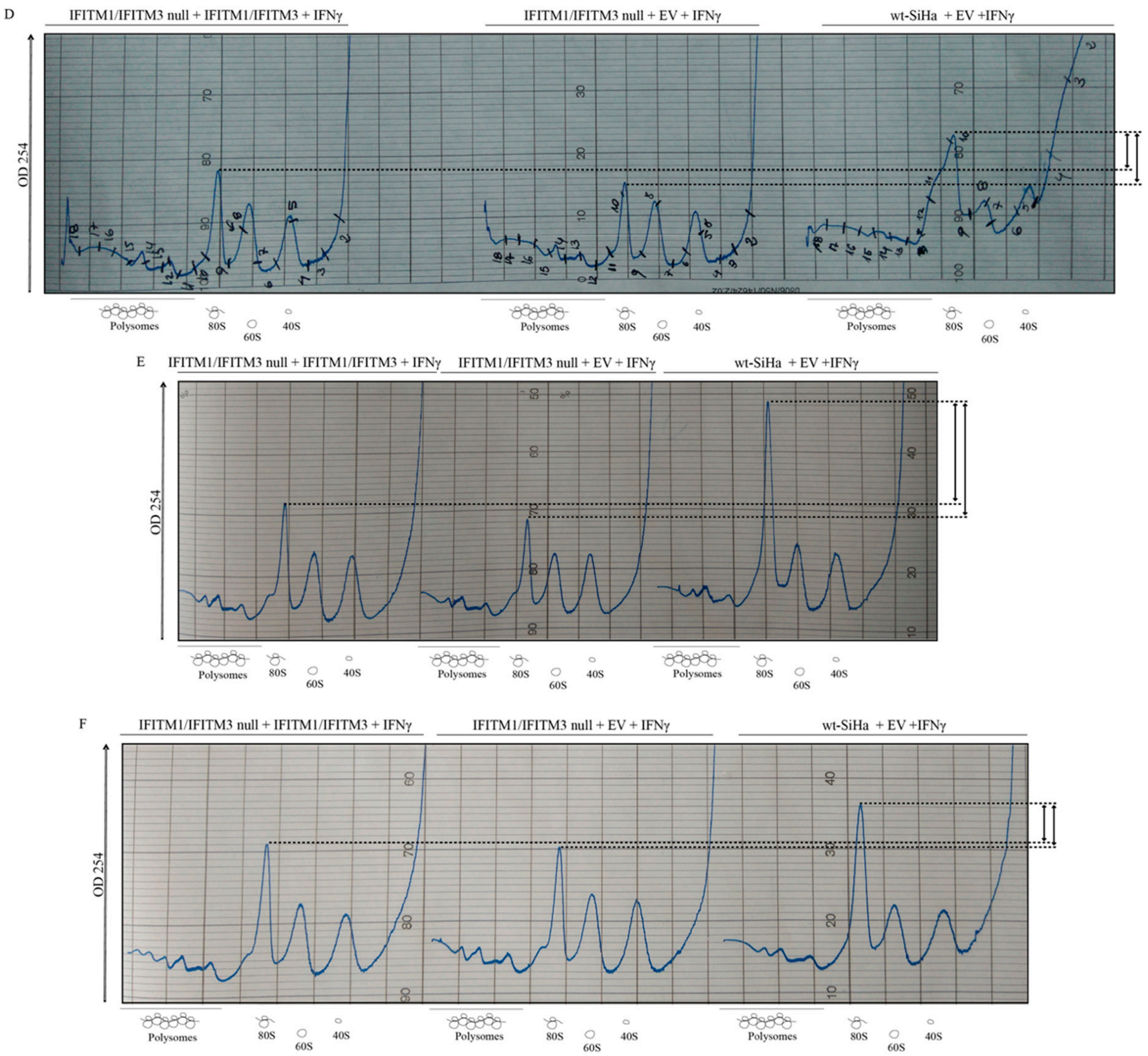

Figure S4. Original scans for the mRNA trace after sucrose density gradient fractionation in six independent replicates. (A–C) Sucrose density gradient was performed in wt-SiHa cells and IFITM1/IFITM3 null cells treated with 100 ng/ml IFNγ for 24 h to activate the IFN-protein synthesis response. A higher 80S peak (A254) was observed in wt-SiHa cells compared to in IFITM1/IFITM3 null cells in all three replicates (indicated with the dashed lines). (D-F) Sucrose density gradient was performed in wt-SiHa cells and IFITM1/IFITM3 null cells treated with 100 ng/ml IFNγ for 24 h. Cells were also transfected with IFITM1 and IFITM3, or the respective empty vector (EV) for 48 h to recover the lower 80S peak observed in the IFITM1/IFITM3 null cells (indicated with the dashed lines).

Table S1. List of proteins enriched in SBP-IFITM1 pull down. The precipitates at both 24 and 48 h transfection times were processed as in the experimental methods. The data in the multiconsensus report are represented, as in column: A. *Accession number*, B. *Description* (gene name). Summary from all analyzed samples. C. ΣCoverage (the number of amino acids in a protein sequence that were found in identified peptides), D. *Σ# Proteins* (number of proteins identified in the protein group; introduced is the master protein), E. *Σ# Unique Peptides* (number of peptides that are unique to a protein group), F. *Σ# Peptides* (the total number of distinct peptides in protein group), G. *Σ# PSMs* (the number of peptide spectrum matches, the total number of spectra used for the identification of the peptides belongs to the protein). 24 h time point; H. XCorr (the goodness of fit of experimental peptide fragments to theoretical spectra created from the sequence *b* and *y* ions)); I *Coverage*, J. # Peptides, K. *#PSM*, and L. Area (under the peak, value used for quantification). 48 h time point; M, XCorr; N *Coverage*, O # Peptides, P. *#PSM*, and Q. Area. The ratio of the relative peak intensities of the heavy to light peptides are highlighted at 24 h (R) and 48 h (U).

Table S2. Generation of RNA seq datasets from the indicated cell lines using CLCBio Genomics workbench 12.0. The fastq sequencing reads were used as the input file and RNA-seq analysis tool was used in the CLCbio Genomics workbench 12.0. All transcript reads detected were taken to generate the final transcript count for each gene. Comparisons of all transcripts were performed for the following cells: nontreated wt-SiHa cells (index 13 fastq files), IFNγ-stimulated wt-SiHa cells (index 14 fastq files), nontreated IFITM1/IFITM3 null cells (index 23 fastq files), and vs IFNγ-stimulated IFITM1/IFITM3 null cells (index 25 fastq files). The output excel file from the software in the columns represent: gene name; chromosome location; region; identifier; wild type SiHa total counts; wild type SiHa RPKM; wild type SiHa TPM; wild type SiHa CPM; wild type SiHa IFNγ total counts; wild type SiHa IFNγ RPKM; wild type SiHa IFNγ TPM; wild type SiHa IFNγ CPM; IFITM1/IFITM3 null total counts; IFITM1/IFITM3 null RPKM; IFITM1/IFITM3 null TPM; IFITM1/IFITM3 null CPM; IFITM1/IFITM3 null IFNγ total counts; IFITM1/IFITM3 null IFNγ RPKM; IFITM1/IFITM3 null IFNγ TPM; IFITM1/IFITM3 null IFNγ CPM.
